# Genetic prediction of modifiable lifestyle factors for erectile dysfunction

**DOI:** 10.1093/sexmed/qfae010

**Published:** 2024-03-18

**Authors:** Yu-Jia Xi, Yi-Ge Feng, Ya-Qi Bai, Rui Wen, He-Yi Zhang, Qin-Yi Su, Qiang Guo, Cheng-Yong Li, Zhen-Xing Wang, Liang Pei, Sheng-Xiao Zhang, Jing-Qi Wang

**Affiliations:** Department of Urology, The Second Hospital of Shanxi Medical University, Taiyuan 030001, China; Shanxi Provincial Key Laboratory of Rheumatism Immune Microecology, Taiyuan, Shanxi Province 030001, China; Key Laboratory of Cellular Physiology at Shanxi Medical University, Ministry of Education, Taiyuan, Shanxi Province 030001, China; Shanxi Provincial Key Laboratory of Rheumatism Immune Microecology, Taiyuan, Shanxi Province 030001, China; Key Laboratory of Cellular Physiology at Shanxi Medical University, Ministry of Education, Taiyuan, Shanxi Province 030001, China; Shanxi Provincial Key Laboratory of Rheumatism Immune Microecology, Taiyuan, Shanxi Province 030001, China; Key Laboratory of Cellular Physiology at Shanxi Medical University, Ministry of Education, Taiyuan, Shanxi Province 030001, China; Shanxi Provincial Key Laboratory of Rheumatism Immune Microecology, Taiyuan, Shanxi Province 030001, China; Key Laboratory of Cellular Physiology at Shanxi Medical University, Ministry of Education, Taiyuan, Shanxi Province 030001, China; Shanxi Provincial Key Laboratory of Rheumatism Immune Microecology, Taiyuan, Shanxi Province 030001, China; Key Laboratory of Cellular Physiology at Shanxi Medical University, Ministry of Education, Taiyuan, Shanxi Province 030001, China; Department of Urology, The Second Hospital of Shanxi Medical University, Taiyuan 030001, China; Shanxi Provincial Key Laboratory of Rheumatism Immune Microecology, Taiyuan, Shanxi Province 030001, China; Key Laboratory of Cellular Physiology at Shanxi Medical University, Ministry of Education, Taiyuan, Shanxi Province 030001, China; Department of Urology, The Second Hospital of Shanxi Medical University, Taiyuan 030001, China; Department of Urology, The Second Hospital of Shanxi Medical University, Taiyuan 030001, China; Department of Urology, The Second Hospital of Shanxi Medical University, Taiyuan 030001, China; Department of Urology, The Second Hospital of Shanxi Medical University, Taiyuan 030001, China; Shanxi Provincial Key Laboratory of Rheumatism Immune Microecology, Taiyuan, Shanxi Province 030001, China; Key Laboratory of Cellular Physiology at Shanxi Medical University, Ministry of Education, Taiyuan, Shanxi Province 030001, China; Department of Rheumatology, The Second Hospital of Shanxi Medical University, Taiyuan 030001, China; Department of Urology, The Second Hospital of Shanxi Medical University, Taiyuan 030001, China

**Keywords:** erectile dysfunction, lifestyle factors, Mendelian randomization, genome-wide association, causal effect

## Abstract

**Background:**

The causal relationship between certain lifestyle factors and erectile dysfunction (ED) is still uncertain.

**Aim:**

The study sought to investigate the causal effect of 9 life factors on ED through 2-sample single-variable Mendelian randomization (SVMR) and multivariable Mendelian randomization (MVMR).

**Methods:**

Genetic instruments to proxy 9 risk factors were identified by genome-wide association studies. The genome-wide association studies estimated the connection of these genetic variants with ED risk (n = 223 805). We conducted SVMR, inverse variance-weighting, Cochran’s Q, weighted median, MR-Egger, MR-PRESSO (Mendelian Randomization Pleiotropy RESidual Sum and Outlier), and MVMR analyses to explore the total and direct relationship between life factors and ED.

**Outcomes:**

The primary outcome was defined as self or physician-reported ED, or using oral ED medication, or a history of surgery related to ED.

**Results:**

In SVMR analyses, suggestive associations with increased the risk of ED were noted for ever smoked (odds ratio [OR], 5.894; 95% confidence interval [CI], 0.469 to 3.079; *P* = .008), alcohol consumption (OR, 1.495; 95% CI, 0.044 to 0.760; *P* = .028) and body mass index (BMI) (OR, 1.177; 95% CI, 0.057 to 0.268; *P* = .003). Earlier age at first intercourse was significantly related to reduced ED risk (OR, 0.659; 95% CI, −0.592 to −0.244; *P* = 2.5 × 10^−6^). No strong evidence was found for the effect of coffee intake, time spent driving, physical activity, and leisure sedentary behaviors on the incidence of ED (All *P* > .05). The result of MVMR analysis for BMI (OR, 1.13; 95% CI, 1.01 to 1.25; *P* = .045) and earlier age at first intercourse (OR, 0.77; 95% CI, 0.56 to 0.99; *P* = .018) provided suggestive evidence for the direct impact on ED, while no causal factor was detected for alcoholic drinks per week and ever smoked.

**Clinical implications:**

This study provides evidence for the impact of certain modifiable lifestyle factors on the development of ED.

**Strengths and limitations:**

We performed both SVMR and MVMR to strengthen the causal relationship between exposures and outcomes. However, the population in this study was limited to European ancestry.

**Conclusion:**

Ever smoked, alcoholic drinks per week, BMI, and age first had sexual intercourse were causally related to ED, while the potential connection between coffee intake, physical activity, recreational sedentary habits, and increased risk of ED needs to be further confirmed.

## Introduction

Erectile dysfunction (ED), a multidimensional male sexual disorder, is the reduplicative incapacity to generate or sustain a penile erection adequate for successful vaginal intercourse to engage in satisfactory sexual intercourse.^1^ This symptom is closely related to vascular disease, endocrine disorders, and neurological and mental health.^2^ According to the European Association of Urology 2021 Guidelines for Male Diseases, the morbidity of ED rose with age, ranging from 12% to 82.9%,^3^ with an average prevalence of 30%.^4^ Therefore, identifying the risk factors of ED is critical and beneficial to developing preventive interventions and reducing the incidence of ED.

Previous observational studies have revealed a large number of modifiable risk factors for ED, covering smoking, alcohol consumption, diet, coffee intake, and psychological disorders.^5^^,^^6^ Physical activity and exercise interventions improve patient-reported ED.^7^ However, some observational studies do not support the association between smoking and alcohol consumption and ED.^8^^,^^9^ Due to their observational nature, the biases from confounders and reverse causality cannot be avoided, so the results varied in different surveys and the causal relationship between modifiable lifestyle factors and ED remains controversial and needs further exploration.

To avoid the bias of observational studies, Mendelian randomization (MR), a popular analytical method, could be used to explore the causal impact of lifestyle factors on ED. MR uses genetic variants as instrumental variables (IVs) and carries 2 strengths minimizing confounding and decreasing reverse causality because genetic variants are randomly allocated at conception and cannot be affected by disease status. Hence, MR analysis can replicate a naturally occurring controlled trial to test causal estimates.^10^ If the IV is linked to both the exposure and the confounder, their effects on the outcome can be estimated jointly using multivariable MR (MVMR).^11^ It is a single-variable MR (SVMR) extension that can directly assess the impact of individual risk factors while avoiding the effects of other potentially related risk factors.^12^

In this study, we included 9 lifestyle factors as exposure factors, including ever smoked, alcoholic drinks per week, body mass index (BMI), earlier age at first intercourse, coffee intake, time spent using computer, number of vigorous physical activity 10+ minutes, number of days per week of moderate physical activity 10+ minutes, and time spent driving, and explored whether there was a causal relationship between exposure and ED through the 2-sample SVMR and MVMR analyses.

## Methods

### Study design

**Figure 1:**
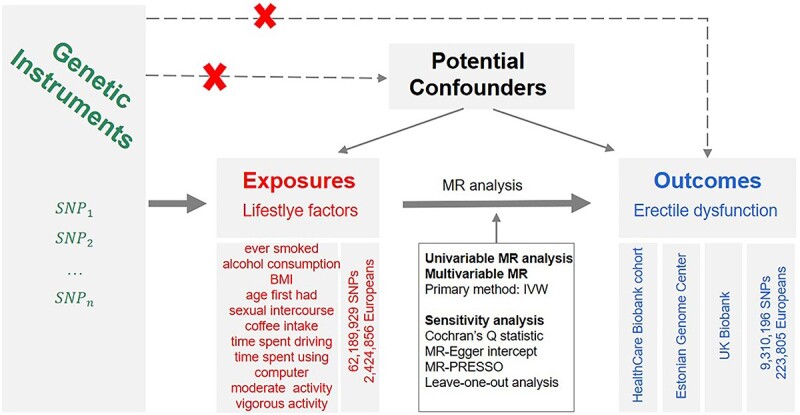
Description of the study design in this 2-sample MR study.

The current study was a 2-sample MR investigation that used data from global genetic consortia. We report this MR study with reference to STROBE-MR (Strengthening the Reporting of Observational Studies in Epidemiology Using Mendelian Randomization).^13^ MR studies must satisfy 3 assumptions, which include (1) genetic variants are robustly related to the risk factor of interest, (2) the absence of confounding factors for the genetic variation-outcome association, and (3) elimination of the restrictive hypothesis (Figure 1).

### Data source

Pooled data associated with ED were extracted from the study conducted by Bovijn et al,^14^ which is currently the most comprehensive genome-wide association study (GWAS) of ED. This study included 6175 patients with ED vs 217 630 control subjects among 223 805 European males based on the hospital-recruited Partners HealthCare Biobank cohort, the UK Biobank (UKBB), and the Estonian Genome Center of the University of Tartu cohort. It was diagnosed using International Classification of Diseases–Tenth Revision codes (N48.4 and F52.2) and medical histories of drug or surgical intervention in ED. A detailed overview of all data sources can be found in Table 1.

**Table 1:** All detailed data sources.

Trait	Consortium	Sample size	Population	Dataset
Alcoholic drinks per week	GWAS and Sequencing Consortium of Alcohol and Nicotine use	335 394	European	ieu-b-73
Ever smoked	MRC-IEU	461 066	European	ukb-b-20 261
Body mass index	Neale Lab	336 107	European	ukb-a-248
Age at first sexual intercourse	MRC-IEU	406 457	European	ukb-b-6591
Coffee intake	MRC-IEU	428 860	European	ukb-b-5237
Vigorous physical activity	MRC-IEU	440 512	European	ukb-b-151
Time spent driving	MRC-IEU	310 555	European	ukb-b-3793
Time spent using computer	MRC-IEU	360 895	European	ukb-b-4522
Moderate physical activity	MRC-IEU	440 266	European	ukb-b-4710
Erectile dysfunction	NA	223 805	European	ebi-a-GCST006956

Abbreviations: GWAS, genome-wide association study; MRC-IEU, Medical Research Council Integrative Epidemiology Unit.

We identified the biggest reported GWAS among people of European ancestry for each lifestyle component. Genetic instruments for alcoholic drinks per week were obtained from the most recent meta-analysis on tobacco and alcoholic drinks per week based on over 30 GWASs of 518 633 individuals of predominantly European ancestry, which measured the effect by the average number of drinks a participant reported drinking each week. For example, if an individual reported 1 to 5 drinks per week, we assumed that they drank 2.5 drinks per week on average.^15^ Summary-level data for ever-smoked phenotypes, earlier age at first intercourse, and coffee intake were retrieved from the GWAS pipeline using PHESANT-derived variables from the UKBB by the Medical Research Council Integrative Epidemiology Unit, which consisted of 461 066 participants (280 508 cases and 180 558 controls) for ever smoked, 406 457 European individuals for earlier age at first intercourse, 440 512 participants for number of days per week of vigorous physical activity 10+ minutes, 310 555 individuals for time spent driving, 360 895 participants for time spent using computer, 440 266 individuals for number of days per week of moderate physical activity 10+ minutes, and 428 860 individuals for coffee intake. GWAS statistics significantly associated with BMI were obtained from Neale Lab with 336 107 European ancestry individuals. Screening details of these risk factor phenotypes were presented in Supplementary Table 2.

### IV selection

Single nucleotide polymorphisms (SNPs) with genome-wide significance (*P* = 5 × 10^−8^) were suggested as IVs. To avoid colinearity, SNPs with linkage imbalance (R^2^ > 0.01 and clump window < 10 000 kb) were excluded, and SNPs with the greatest influence on related traits were retained. SNPs with low allele frequency (Minor Allele Frequency (MAF) < 0.01) were deleted. Furthermore, to eliminate the bias of weak IVs, the following formula was used to calculate the F-statistic of SNPs: F = R2(N-K-1)/[K(1-R2)]. The values of F statistics denoted the strength of IVs, with F statistics >10 considered powerful IVs.

### Statistical analysis

For SVMR analysis, we employed inverse variance–weighted (IVW) MR as the primary method, which was primarily utilized for fundamental causal estimates and delivered the most accurate findings when all of the identified SNPs were genuine IVs. However, the IVW method assumes the validity of all genetic variants used as IVs. To ensure the robustness of our results, additional sensitivity analyses were performed using complementary MR-Egger and weighted median–based regression approaches, which could offer more reliable estimations in a larger range of scenarios.

The positive results from SVMR were included in the MVMR analysis. We account for both measurable and unmeasured pleiotropy using the MVMR extension of the IVW MR approach in each GWAS meeting our SVMR selection criteria.

### Sensitivity analysis

Heterogeneity was quantified using Cochran’s Q statistic. If the *P* value of the Cochran Q test was <.1, heterogeneity was observed. The MR-Egger regression model discovered possible pleiotropy with *P* for intercept <.05.^16^ MR-PRESSO (Mendelian Randomization Pleiotropy RESidual Sum and Outlier) was applied to detect and eliminate potential outliers to re-estimate the original exposure-outcome relationship (*P* < .05).^17^

All statistical analyses were performed using the TwoSampleMR, MR-PRESSO, and mr.raps packages in R software version 4.1.2 (R Foundation for Statistical Computing).

#### Ethics

As this study was a secondary analysis based on public summary-level GWAS datasets. All subjects in the original studies have provided informed consent, so further ethical approval is not required.

## Results

### Genetic instruments

SNPs in lifestyle factors and ED were extracted for exposures, which met the genome-wide threshold. The F-statistic of each SNP was greater than10. Details of the identified IVs relating to ED are presented in Supplementary Table 1.

### SVMR analyses

**Figure 2:**
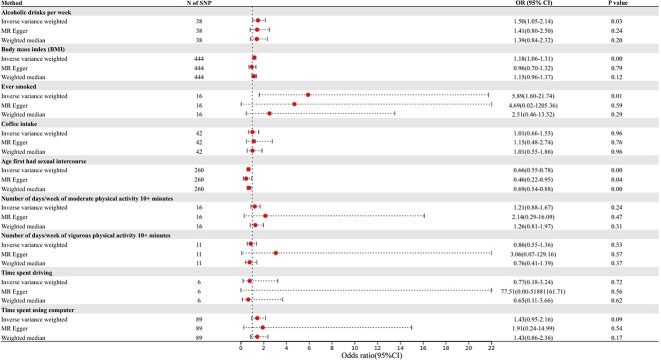
Odds ratios (ORs) and 95% confidence intervals (CIs) for the effect of 9 life factors on erectile dysfunction estimated using the single-variable Mendelian randomization (MR). N: Number of SNPs used in the MR.

**Figure 3:**
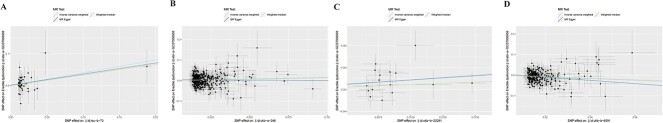
Scatter plot for the life factors with significant influence on erectile dysfunction. (A) Alcoholic drinks per week; (B) body mass index; (C) smoking history; (D) age at first intercourse.

**Figure 4:**
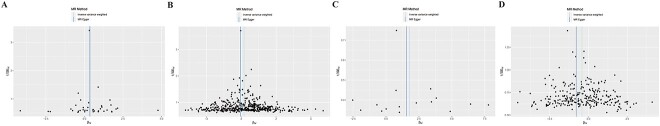
Funnel plot for the life factors with significant influence on erectile dysfunction. (A) Alcoholic drinks per week; (B) body mass index; (C) smoking history; (D) age at first intercourse.

From the SVMR analyses (Table 2; Figures 2, 3, and 4; Supplementary Figure 1), the genetic prediction of the IVW method showed that more alcoholic drinks per week (odds ratio [OR], 1.50, 95% confidence interval [CI], 1.05-2.14; *P* = .03), BMI (OR, 1,18; 95% CI, 1.06-1.31; *P* < .01), and longer smoking history (OR, 5.89; 95% CI, 1.60-21.74; *P* < .01) could increase the risk of ED, whereas strong genetic evidence that earlier age at first intercourse (OR, 0.66; 95% CI, 0.55-0.78; *P* < .01) might elevate the risk of it. The results of MR-Egger and the weighted median method were substantially similar to those of IVW. In sensitivity analysis, the results of Cochran’s Q test and MR-Egger regression showed no significant heterogeneity and horizontal pleiotropy in SVMR analysis (P > .05). Additionally, the results of the leave-one-out sensitivity tests demonstrated that the removal of either SNP did not change the causal relationship (Supplementary Figure 2).

**Table 2:** Causal relationships of life factors on ED by univariable Mendelian randomization.

	Outcome	Method	Heterogeneity test		MR-Egger pleiotropy test	
Exposure			Q	*P* value	Intercept	*P* value
Alcoholic drinks per week	ED	Inverse variance weighted	30.370	.77	0.001	.81
		MR-Egger	30.310	.74		
		Weighted median				
Body mass index	ED	Inverse variance weighted	445.689	.46	0.004	.18
		MR-Egger	443.869	.47		
		Weighted median				
Ever smoked	ED	Inverse variance weighted	18.323	.25	0.002	.93
		MR-Egger	18.314	.19		
		Weighted median				
Coffee intake	ED	Inverse variance weighted	32.166	.84	−0.002	.74
		MR-Egger	32.058	.81		
		Weighted median				
Age at first intercourse	ED	Inverse variance weighted	275.453	.23	0.006	.31
		MR-Egger	274.352	.23		
		Weighted median				
Number of days per week of moderate physical activity 10+ min	ED	Inverse variance weighted	11.395	.72	−0.019	.58
		MR-Egger	11.081	.68		
		Weighted median				
Number of days per week of vigorous physical activity 10+ min	ED	Inverse variance weighted	10.426	.40	−0.037	.52
		MR-Egger	9.935	.36		
		Weighted median				
Time spent driving	ED	Inverse variance weighted	1.745	.88	−0.056	.54
		MR-Egger	1.286	.86		
		Weighted median				
Time spent using computer	ED	Inverse variance weighted	115.896	.02	−0.004	.78
		MR-Egger	115.788	.02		
		Weighted median				

Abbreviations: CI, confidence interval; ED, erectile dysfunction; OR, odds ratio.

### MVMR analyses

**Figure 5:**
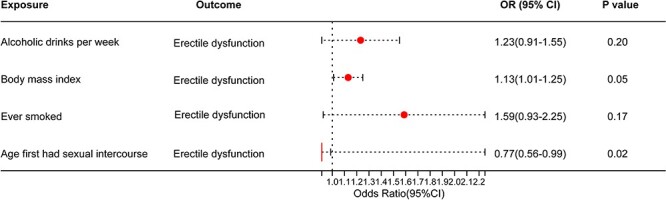
Odds ratios (ORs) and 95% confidence intervals (CIs) for the effect of 6 life factors on erectile dysfunction estimated using the multivariable Mendelian randomization (MR). N indicates the number of single nucleotide polymorphisms used in the MR.

Positive results in SVMR, which included alcoholic drinks per week, BMI, smoking history, and age at first intercourse, were assessed together in MVMR, only BMI (OR, 1.13; 95% CI, 1.01-1.25; *P* = .05) and age at first intercourse (OR, 0.77; 95% CI, 0.56-0.99; *P* = .02) retained a robust, potentially causal relationship with ED (Table 3, Figure 5). The estimates for alcoholic drinks per week (OR, 1.23; 95% CI, 0.91-1.55; *P* = .20) and smoking history (OR, 1.59; 95% CI, 0.93-2.25; *P* = .17) were weakened substantially (Table 3).

**Table 3:** Causal relationships of specific life factors on ED estimated by multivariable Mendelian randomization.

Exposure	Outcome	OR (95% CI)	*P* value
Alcoholic drinks per week	ED	1.23 (0.91-1.55)	.20
Body mass index	ED	1.13 (1.01-1.25)	.05
Ever smoked	ED	1.59 (0.93-2.25)	.17
Age first had sexual intercourse	ED	0.77 (0.56-0.99)	.02

Abbreviations: CI, confidence interval; ED, erectile dysfunction; MR, Mendelian randomization; OR, odds ratio.

## Discussion

This study represents the first attempt to investigate the association between various life factors and ED using a causal framework provided by MR analysis. Our findings revealed significant relationships between smoking history, drinking history, BMI, and age at first intercourse with an increased likelihood of experiencing ED, while no substantial evidence was found to support the effect of the remaining 5 lifestyles on the incidence of ED.

In our study, current or past regular or occasional smokers were categorized as ever smoked. The deleterious effects of smoking on ED are well established.^18^ The underlying mechanisms could be vascular and endocrinological pathways. One of the smoke-induced vascular damages is that smoking could persistently induce endothelial dysfunction (EnD) by increasing oxidative stress.^19^ The most important physiological mechanism of penile erection is nitric oxide diffusing from the nerve or endothelial cells. Thus EnD caused by smoke could lead to ED.^20^ The endocrinological pathways are related to the hypothalamus-pituitary-testis axis, which is active in current smokers with higher multiple hormones, including testosterone. The hyperandrogenic environment could facilitate smoking behavior.^21^ Therefore, a vicious circle formed.

A significant relationship between alcohol consumption and ED was observed in this study, which is consistent with previous studies.^22^ We used drinks per week as a phenotype of alcohol intake in the research, which is defined as the average number of drinks a participant reported drinking each week, aggregated across all types of alcohol. If a study recorded binned response ranges, we used the midpoint of the range. For example, if an individual reported 1 to 5 drinks per week, we assume that they drank 2.5 drinks per week on average.^15^ Animal experiments have shown that^22^ chronic exposure to alcohol reduces the relaxation response of the corpus cavernosum in vitro through neurogenic and endothelial pathways while attenuating the physiological process of increased pressure in the corpus cavernosum caused by bioelectrical conduction in vivo. Besides, frequent use of alcohol reduces libido and makes it difficult for men to achieve erections or orgasms.^23^

High BMI has been proven to be associated with ED in previous studies.^24^^,^^25^ BMI is constructed from height and weight measured during the initial assessment center visit and an average increase of 0.1 BMI units per BMI-increasing allele, equivalent to 260 to 320 g for an individual 160 to 180 cm in height.^26^ Adipose tissue dysfunction and associated metabolic abnormalities contribute to EnD.^27^^,^^28^ Endocrinologically, adipocytes highly express aromatase, which converts testosterone to estradiol, leading to testosterone depletion, which ultimately may exacerbate the progression of ED, as testosterone is a crucial regulator of nitric oxide synthase expression inside the penis.^29^ Thus, obesity could lead to decreased or even loss of erectile function.

Few studies have examined the association between age at first sexual intercourse and ED, the causal relationship revealed in this study could be caused by psychological factors. In our study, age first had sexual intercourse was filtered by touchscreen questions “What was your age when you first had sexual intercourse? (Sexual intercourse includes vaginal, oral or anal intercourse.)” It has been proved that there is a link between ED and depression.^30^ Early sexual behavior and lack of sexual knowledge easily lead to mental tension and sexual failure, resulting in male psychological disorders.

In this study, no effect of coffee intake and physical activity on the incidence of ED. The study has shown that coffee intake has no significant correlation with ED.^31^ Although there is no strong evidence of an association between physical activity and ED, several common lifestyle factors, such as the absence of physical exercise, have been shown to be linked to the development of ED.^32^ The present studies suggested that physical activity and exercise interventions improve patient-reported ED, particularly aerobic exercise with moderate-to-vigorous intensity.^7^^,^^33^ Therefore, a lack of physical activity can still lead to ED.

In the UKBB, the largest database for this study, the prevalence of ED was 1.53% (n = 3050 of 199 352), which is much lower than data from the European Male Ageing Study (EMAS). It reports that approximately 19% of males between 50 and 59 years of age report having moderate-to-severe ED. Possible reasons for this discrepancy are as follows. Subjects in the UKBB self-reported White ethnicity and in EMAS were recruited from population registers in 8 European centers. In addition, the characteristics of the participants in the 2 studies differed, with the median age of men in the Partners Health Care Biobank cohort being higher in the UKBB (65 years, compared with 59 years in the UKBB and 42 years in the Estonian Genome Center of the University of Tartu) and the mean age of EMAS participants being 60 years. Meanwhile, the UKBB’s “healthy volunteers” were subject to selection bias,^34^ were the UKBB’s lack of primary care data availability, and cross-cultural differences, including a “social expectation” bias.^35^^,^^36^ However, the UKBB study also notes that although prevalence may not be representative of the general population, the assessment of exposure-outcome relationships is valid.

There are several advantages to this study. First, the MR analysis provided reliable insights into causal relationships between risk factors and disease outcomes, reducing potential confounding.^37^ Second, multiple MR analyses rely on orthogonal assumptions to assess the validity of MR assumptions and increase the credibility and robustness of the results.^38^ Third, the ED dataset used in this study was defined as self- or physician-reported ED, or using oral ED medication, or a history of surgery related to ED, which was accurate and reliable. However, limitations are also noted in this study. For instance, the overlap of UKBB participants in many GWAS datasets may bias the estimates, but they are still valid. And with the use of large GWAS datasets and MR analysis methods, any bias is likely to be minimal.^39^ In addition, the population in this study was limited to European ancestry, which prevents the generalization of the causality to other lineages. Moreover, ED is an androgenetic disease. Although the dataset is adjusted for gender, the potential bias of gender effect still exists. Finally, about ever smoke, some literature has examined the relationship between smoke and other comorbidities such as dyslipidemia and atherosclerosis, which are also genetically mediated.^40^^,^^41^ We mainly investigate the association between smoking and ED, and as for the impact of smoking volume on ED, based on this GWAS data, we cannot determine the specific smoking volume of each smoker. More research might be needed in the future to explore the association between smoking quantity and the occurrence of ED, as well as the potential mechanisms involved.

## Conclusion

This study adds to the growing body of evidence supporting the impact of certain modifiable lifestyle factors on the development of ED. The findings have implications for public health initiatives and strategies aimed at promoting healthier lifestyles to reduce the burden of ED.

## Supplementary Material

STROBE-MR-checklist_qfae010

Supplementary_Figure_1_qfae010

Supplementary_Figure_2_qfae010

Supplementary_Table_1_Instrumental_SNPs_from_lifestyle_Factors_GWASs_qfae010

Supplementary_Table_2_Phenotypic_definition_specification_qfae010

## Data Availability

All data used in this study are publicly available.
