# Supplementary figures and images for: Genetic prediction of modifiable lifestyle factors for erectile dysfunction
Source: Sex Med. 2024 Mar 18;12(1):qfae010. doi: 10.1093/sexmed/qfae010 (PMC10949036; doi:10.1093/sexmed/qfae010)

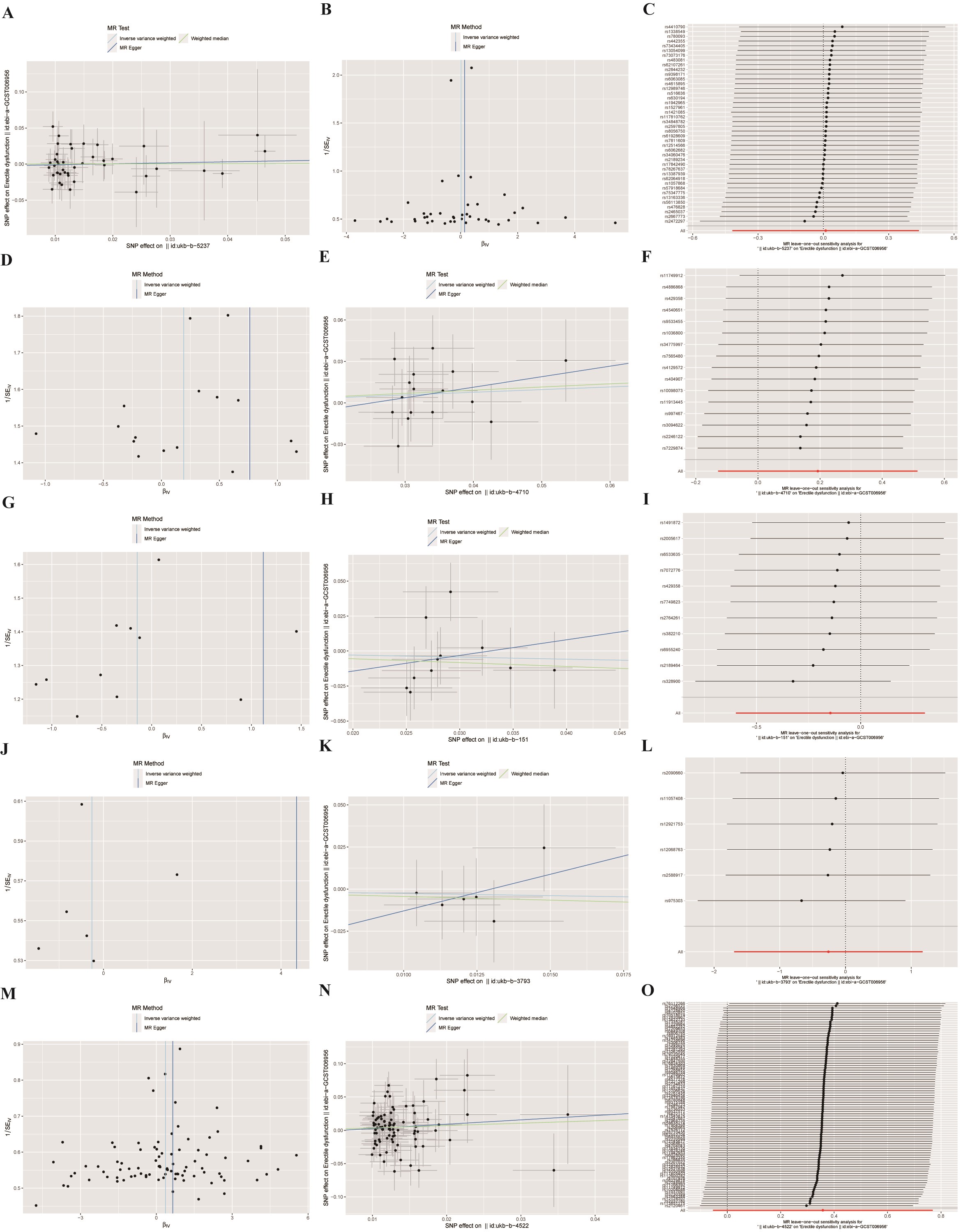

Supplement: Supplementary_Figure_1_qfae010 [file supplementary_figure_1_qfae010.jpeg]

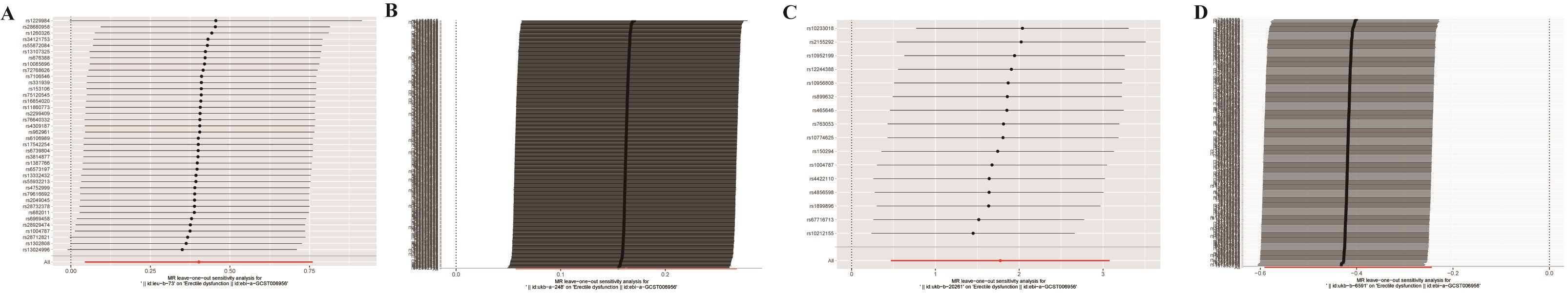

Supplement: Supplementary_Figure_2_qfae010 [file supplementary_figure_2_qfae010.jpeg]
